# Aquatic Ecosystems of the Anthropocene: Limnology and Microbial Ecology of Mine Pit Lakes

**DOI:** 10.3390/microorganisms9061207

**Published:** 2021-06-03

**Authors:** Melanie L. Blanchette, Mark A. Lund

**Affiliations:** Mine Water and Environment Research Centre (MiWER), School of Science, Edith Cowan University, 270 Joondalup Drive, Joondalup 6101, Australia; m.lund@ecu.edu.au

**Keywords:** Bowen, Hunter, 16S, SILVA, Illumina, PERMANOVA, thermistor, stratification, deep lake, UniFrac

## Abstract

Mine pit lakes (‘pit lakes’) are new aquatic ecosystems of the Anthropocene. Potentially hundreds of meters deep, these lakes are prominent in the landscape and in the public consciousness. However, the ecology of pit lakes is underrepresented in the literature. The broad goal of this research was to determine the environmental drivers of pelagic microbe assemblages in Australian coal pit lakes. The overall experimental design was four lakes sampled three times, top and bottom, in 2019. Instrument chains were installed in lakes and measurements of in situ water quality and water samples for metals, metalloids, nutrients and microbe assemblage were collected. Lakes were monomictic and the timing of mixing was influenced by high rainfall events. Water quality and microbial assemblages varied significantly across space and time, and most taxa were rare. Lakes were moderately saline and circumneutral; Archeans were not prevalent. Richness also varied by catchment. Microbial assemblages correlated to environmental variables, and no one variable was consistently significant, spatially or temporally. Study lakes were dominated by ‘core’ taxa exhibiting temporal turnover likely driven by geography, water quality and interspecific competition, and the presence of water chemistry associated with an artificial aquifer likely influenced microbial community composition. Pit lakes are deceptively complex aquatic ecosystems that host equally complex pelagic microbial communities. This research established links between microbial assemblages and environmental variables in pit lakes and determined core communities; the first steps towards developing a monitoring program using microbes.

## 1. Introduction

The impact of mining on the landscape is a permanent legacy of industrialisation. New lakes created by flooding abandoned open-cut coal mines with groundwater, surface water and rainwater occur on every inhabited continent [1] although the exact number is unknown [2]. These lakes of the Anthropocene (mine pit lakes or ‘pit lakes’) diverge widely according to morphology and water quality. However, the technological advances of modern mining have increased the size of open-cut pits (and therefore, pit lakes) far beyond the first hand-hewn coal strip mines of the midwestern United States [3], or the 2000-year-old Roman gold pits in northwest Spain [4]. The depth of modern mine pits is often measured in the hundreds or thousands of meters, such as the Kalgoorlie Super Pit in Western Australia (480 m) or the Bingham Canyon Copper Mine in the US state of Utah (1200 m).

As aquatic ecosystems of the Anthropocene [5], pit lakes are prominent in the public consciousness in terms of environment, history and culture [6,7]. However, their broader ecology has not been well represented in the primary source literature as they are perceived as industrial and government ‘problems’ to treat [8] rather than new ecosystems [9,10]. Few natural co-occurring analogues to modern pit lakes exist [10,11]; bare canyons devoid of organic matter, filled with water of varying qualities, many of which occur in regions with little surface water. The most apt analogy to pit lakes are asteroid or crater lakes, particularly in terms of their oligotrophic status [12,13], minimal catchments and high rocky riparian walls [11].

The water quality of pit lakes can vary substantially and is a function of surrounding geology and catchment interaction [14]. As products of the mining industry, the aquatic microbiology of pit lakes is often viewed through the lens of the microbially-mediated biogeochemistry of ‘extreme’ environments. Improving and identifying the activity of sulfate-reducing bacteria (SRBs) is commonly a key focus, as many of the most famous pit lakes are highly acidic and metalliferous [15]. However, not all open-cut lakes are “giant cups of poison” [16], and even the most notorious have shown improvement [17]. Pit lakes can provide ecosystem services, such as hosting ‘superior’ chemical and biological water quality relative to adjacent natural waters [10] or replacing lost wetlands as nutrient filters [18].

The simplest yet most critical question in microbial ecology is “who is there?” [19]. 16S high-throughput sequencing has facilitated a deeper and more accurate identification of the microbial assemblages of pit lakes resulting in discovery of, for example, novel “poly-extremophiles” [20] capable of withstanding low-pH, high salinity, multiple extreme concentrations of metals and oligotrophy [21,22,23]. The logical extension to the question ‘who is there?’ would be “why are they there?” Exploring correlations between the environmental variables and microbial assemblages is the first step towards understanding the broad microbial ecology of pit lakes. Establishing clear, defensible and repeatable links between the environment and microbial assemblages is a key part in implementing microbes as ecological indicators in pit lakes [24].

The overall goal of this research was to determine the environmental drivers of pelagic microbe assemblages in Australian coal mine pit lakes. We approached the research using traditional techniques in limnology such as attention to lake morphology, fit-for-purpose in situ continuous monitoring, measures of water quality and chemistry, and an experimental design that sampled microbial assemblages across space and time and at the surface and bottom of lakes. We tested the null hypotheses of no significant difference in water quality and microbe assemblages over space, time and depth (top and bottom of the lake), and found no correlation of water quality variables with microbe assemblages.

## 2. Materials and Methods

### 2.1. Study Area

**Figure 1:**
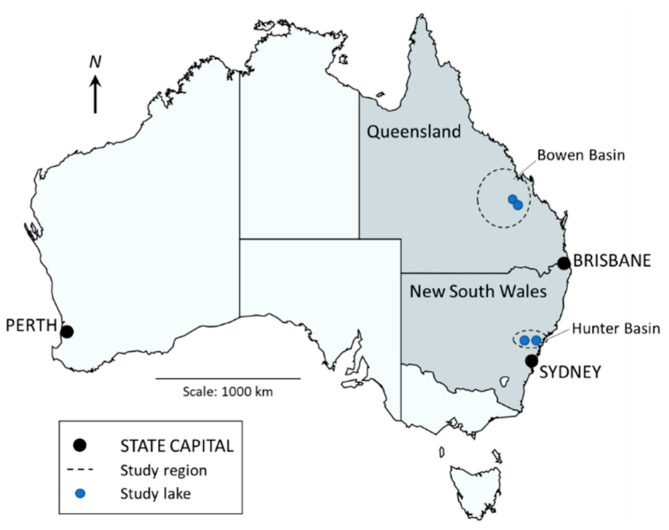
Map of Australia showing approximate location of study lakes in the Bowen Basin, Queensland and the Hunter Basin, New South Wales.

Four Australian mine pit lakes were sampled in this study, two from the Hunter Basin (21,500 km^2^) in the state of New South Wales, and two from the Bowen Basin (60,000 km^2^) in the state of Queensland (Figure 1). The target resource for all mines was coal. The Hunter Basin is in Köppen climate type Cfa (humid subtropical and mild, with no true dry season and with hot summers) [25]. By area, the most widespread land use in the Hunter Basin was stock grazing (39.3%), followed by ‘conservation’ (23.2%), ‘other minimal use’ (16.8%), urban (7.8%), cropping/horticulture (4.4%), forestry (4.3%), water infrastructure (3.0%) and mining (1.1%) [26].

The Bowen Basin sits within Koppen climate type of BSa (arid steppe, with hot summers) and rainfall is summer-dominant (72–29%) [25]. Sourcing percent land use by activity was not possible for the Bowen Basin proper, although the Bowen Catchment sits within the Burdekin Catchment, and the majority of the land use by area in the Burdekin was ‘grazing in native vegetation’ (90%), followed by ‘nature conservation’ (3%). Urban use was 0.18% and mining is 0.11% of the Burdekin Catchment [27], although most of the coal reserves are outside the catchment and therefore mining as an area of use was likely higher.

**Figure 2:**
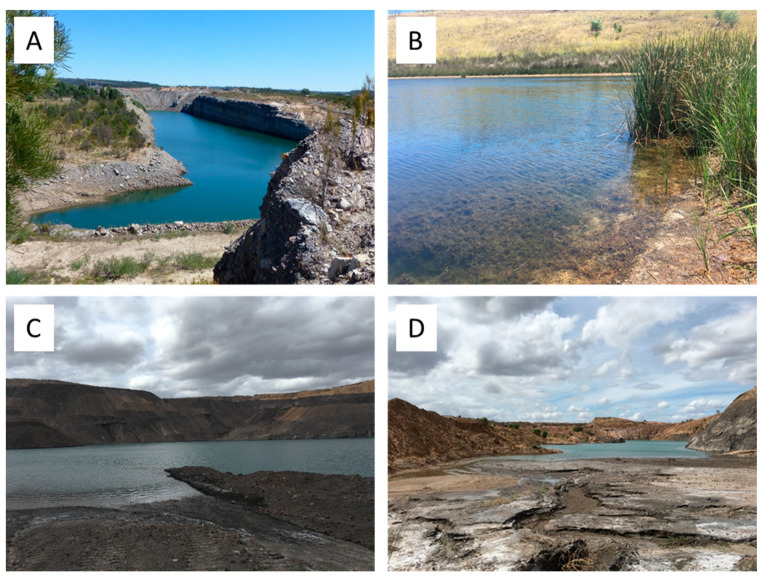
Photographs of Australian mine pit lakes. (**A**) Hunter Lake 1 (HL1), (**B**) Hunter Lake 2 (HL2), (**C**) Bowen Lake 1 (BL1) and (**D**) Bowen Lake 2 (BL2).

Hunter Lake 1 (HL1; Figure 2A) had undergone no rehabilitation and was used for storage of water that entered the northern end of the lake via a wetland area; water was removed as required by mining operations. At the time of sampling in 2019, the lake was approximately 20 years old, 26 m deep and had a surface area of 152,027 m^2^. The southern end of the lake was slowly being filled with tailings via a series of dams.

Hunter Lakes 1 and 2 are approximately 200 km apart in a geographical line. Hunter Lake 2 (HL2; Figure 2B) was a small (7966 m^2^) and shallow (<5 m depth) lake that was physically contoured around the edges by the mining company to form littoral areas. The immediate catchment and littoral area had been planted with local taxa that were well-established and aquatic plants were abundant in the lake. The lake was mined from the late 1980s to the early 2000s, with rehabilitation and contouring occurring shortly after the cessation of mining.

Bowen Lake 1 (BL1; Figure 2C) was still in the process of filling at the time of sampling and had only reached approximately 1/3 final size at 27 m depth and 11.4 ha. BL1 was less than 5 years old, with no rehabilitation. The pit was unlikely to receive groundwater with the lake being created through surface water, precipitation and occasional pumping according to mining requirements.

Bowen Lake 2 (BL2; Figure 2D) was approximately 1 km from BL1. BL2 was also unrehabilitated and approximately 12 m deep with a surface area of 2.6 ha. Approximately 10 years old, BL2 received water only from surface runoff and rainfall. BL2 was occasionally used for operational water pumping or storage.

### 2.2. Continuous Lake Monitoring—Instrument Chains

**Scheme 1:**
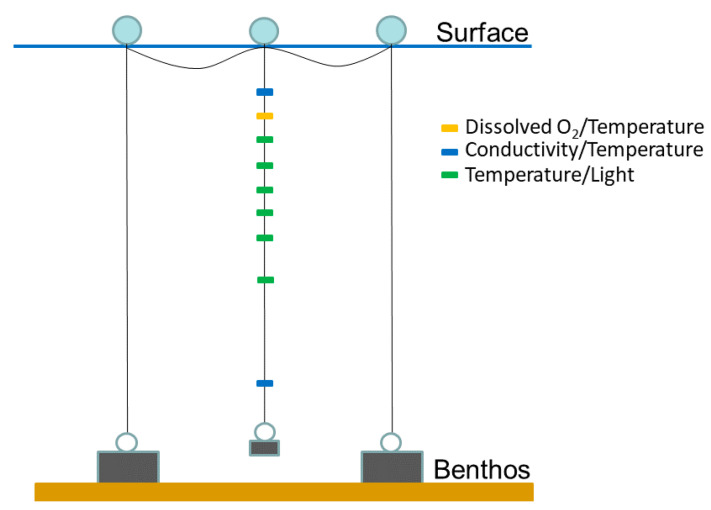
In situ continuous monitoring instrument chains in pit lakes. Light blue circles are buoys, dark rectangles and white circles are weights. (N.b., number of temperature sensors varied depending on chain length).

Instrument chains (Scheme 1) were installed in lakes for the duration of 2019 (beyond the sampling timeframe) to understand stratification in mine pit lakes. Chains were installed in all lakes except HL2 as it was less than 5 m deep and was therefore unlikely to stratify for prolonged periods. Here we describe all data probes on the instrument chain, but for the purposes of this research, temperature is the only data reported. The in situ instrument chains were installed at the deepest point in each lake and off-the-shelf loggers monitored data on an hourly basis. Building on the authors’ previous research using instrument chains in mine pit lakes [28,29] the chains were suspended between two weighted buoys in lakes approximately 20 m apart connected by a surface rope (Scheme 1). The instrument chain was suspended with a small weight at the bottom and a buoy at the top such that it could move somewhat, yet remain taut in the water column. Sensors were attached to the middle rope with cable ties. Sensors were: Hobo Temp-Light Data Logger (UA-002-64 and MX2202), HOBO Water Level Logger—4 m U20L-01, HOBO 76 m Depth Water Level Data Logger (U20-001-03), HOBO Conductivity-Salinity Data Logger (U24-001) and HOBO Dissolved Oxygen Logger—U26-001 (ONSET, Cape Cod, MA, USA). Depth measurements were corrected using air pressure (measured using a U20L-01 depth sensor) as per Baird [30] in HOBOware Pro v3.7 (ONSET, Cape Cod, MA, USA). Due to the relatively limited range of the depth sensor, it was mounted at the base of a separate buoy in shallower water in each lake.

Determination of lake water quality consisted of in situ ‘water quality measurements’ at the top and bottom lake waters and samples collected for ‘water chemistry analysis.’ In situ water quality measurements were: temperature, pH, dissolved oxygen (luminescent), electrical conductivity standardized at 25 °C (EC) and oxidation–reduction potential (ORP, platinum electrode) and percent oxygen (Hydrolab Datasonde DS5 multiparameter instrument (Hach, Austin, TX USA)) after the recommendations of Gammons and Tucci [31]. Three in situ water quality measurements were collected at the top and bottom of each lake (*n* = 6 per lake and time) at the same locations where microbe collection occurred. Locations were approximately equidistant over the lake and included the deepest areas.

The samples collected for water chemistry analysis (see below) were processed in the field and lab according to [23]. One sample was collected at the top and one at the bottom of each lake, based on a composite of the three sites within each lake for a total *n* of 24 per time. An unfiltered aliquot was frozen (−12 °C) for determination of TN and TP following persulphate digestion, and another frozen aliquot was thawed and filtered (0.5 µm Metrigard GF, Pall, USA) for determination of Cl^−^ using ion chromatography (Methrohm, Switzerland), nitrate/nitrite (NOx-N), filterable reactive phosphorous (FRP-P) and ammonia (NH_3_-N) on a Lachat autoanalyser (Hach, USA), and DOC (measured as non-purgeable organic C) using a total carbon analyser (Shimadzu, Japan). A second filtered aliquot was acidified with nitric acid to pH < 2 then stored at 4 °C for determination of metals, metalloids and S by ICP-AES/MS. All methods were as per [30] and were conducted at the Edith Cowan University Analytical Chemistry Centre.

### 2.3. Sample Collection

Water samples for water chemistry analysis (metals and nutrients—see above) and identification of pelagic microbes were collected from pit lakes either via hand, pump or Kemmerer bottle. Upon collection, samples were split in half where appropriate; one part for chemistry analysis and one part for identification of microbes. The method of collection was dependent on sample type (lake top or bottom), overall lake depth or mine site operational requirements.

At all lakes, surface water samples were manually collected 0.2 m below the surface using a new HDPE 2 L bottle (Silverlock Packaging, Canning Vale, Western Australia 6155) after being rinsed three times with target (sample) water prior to collection. At shallow HL2, bottom water 0.5 m above the sediment was collected using a Teflon trace metal Kemmerer bottle (Wildco, FL, USA) [32]. In ‘deep’ lakes (BL1, BL2 and HL1) bottom water samples were collected with a 12 V bilge pump (2088-732-244, Shurflo, Cypress, CA, USA) with a 20 m weighted potable 12 mm hose. Prior to sample collection, the pump ran for several minutes to replace all the water in the tube. Maximum sample depth was limited by the length of the pump, but at all lakes pump length was sufficient to reach the hypolimnion, if present.

In the field, water was filtered (Durapore membrane filters, 0.22 μm, Merck Millipore) under gentle vacuum pressure in a plastic filter tower until filter clogged. Therefore, samples did not represent the same volume of water collected but rather a similar mass of material on filters. All equipment was washed with 70% ethanol in the field between sites to avoid cross contamination. All filters were chopped into smaller fragments using new sterile petri dishes and razor blades per sample to facilitate DNA extraction and stored in new plastic tubes (Eppendorf) at −20 °C during transport (as far as practicable) and then at −80 °C until transfer to the Australian Genome Research Facility (AGRF) for DNA extraction, PCR, DNA sequencing and taxonomic identification.

### 2.4. Microbial Analysis

DNA was extracted from filters using a DNeasy PowerLyzer PowerSoil Kit (Qiagen). Briefly, PCR amplicons were generated using the 16S V3/4 primers (341F: CCTAYGGGRBGCASCAG and 806R: GGACTACNNGGGTATCTAAT) and all samples were amplified using 29 cycles (initialised at 95 °C for 7 min, disassociated at 94 °C for 30 s, annealed at 50 °C for 60 s, extension at 72 °C for 60 s, finished at 72 °C for 7 min) with Invitrogen Platinum SuperFi PCR Master Mix (ThermoFisher Scientific) for the primary PCR. A secondary PCR to index the amplicons was performed with Platinum SuperFi II PCR Master Mix (ThermoFisher Scientific). The resulting amplicons were measured by fluorometry (Invitrogen Picogreen) for a minimum quality control requirement of 0.20 ng µL^−1^ of usable PCR product to generate a sequencing output guarantee of 10,000 raw reads and normalised. The eqimolar pool was then measured by TapeStation assay (Agilent Technologies) and Qubit 4 fluorometer (ThermoFisher Scientific) followed by sequencing on the Illumina MiSeq (San Diego, CA, USA) with 2 × 300 base pairs paired-end chemistry.

Diversity profiling analysis was performed with QIIME 2 2019.7 [33]. The demultiplexed raw reads were primer trimmed and quality filtered using the ‘cutadapt’ plugin followed by denoising with DADA2 (Divisive Amplicon Denoising Algorithm 2 [34]) (via q2-dada2). Using QIIME 2 2019.7, taxonomy was assigned to amplicon single variants (ASVs) using SILVA [35]. Samples were rarefied at 17,000 reads for calculating dissimilarity between samples using weighed UniFrac metrics.

### 2.5. Data Analysis

The overall experimental design for pelagic microbes was four lakes sampled three times, top and bottom, in 2019 (February, May and August). The total sample pelagic microbe *n* was 69 (BL1; *n* = 18, BL2; *n* = 18, HL1; *n* = 18, HL2; *n* = 15). HL2 was missing three samples in February due to handling errors.

Data analysis for water quality and microbe assemblage data followed [23]. Briefly, water quality and microbe assemblage data (amplicon single variant data or ‘ASV’ data [34]) were ordinated to illustrate multivariate patterns and correlated against ordination axes. Water quality data were ordinated with principal components analysis (PCA) and ASV data were ordinated with nonmetric multidimensional scaling (NMDS). Correlations were first tested for significance before relationships with specific variables were explored. Hypotheses were tested using PERMANOVA in PRIMER-e [36]. Instrument chain data were collated and presented using Surfer™ (GOLDEN SOFTWARE, LLC, Golden, Colorado USA), and heat map data showing relative abundances of microbes was presented using Orange Data Mining™ [37].

Prior to hypothesis testing, water quality parameters that had all values below detection (Hg and total P) were removed; other parameters with values below the reportable limit but above zero were assigned a number equal to half the detection limit [38]. Water quality data were normalised and the Euclidean distance measure was used for hypothesis testing. Lake, time and depth (top or bottom waters) were fixed factors (9999 permutations, significant *p* < 0.05). Pseudo-F is reported as F [36].

Hypothesis tests on ASV weighted (abundance and presence/absence) UniFrac genetic distance data were also conducted with a PERMANOVA in Primer-e (factors, permutations and significance as per water quality). Correlations between ASV data and water quality were analysed using RELATE (Spearman’s rank *r_s_*, *p* < 0.05; 9999 permutations) with a Pearson’s correlation in PRIMER-e. Pelagic microbe (ASV) taxonomic richness (*S*; common taxa relative abundance >1.0 only) was determined as mean *S* per sample, and was calculated using Kruskall–Wallis one-way ANOVA on ranks in SigmaPlot 13.0 with a Dunn’s method pairwise test (Systat Software, Inc., San Jose, CA, USA).

## 3. Results

### 3.1. Continuous Lake Monitoring

**Figure 3:**
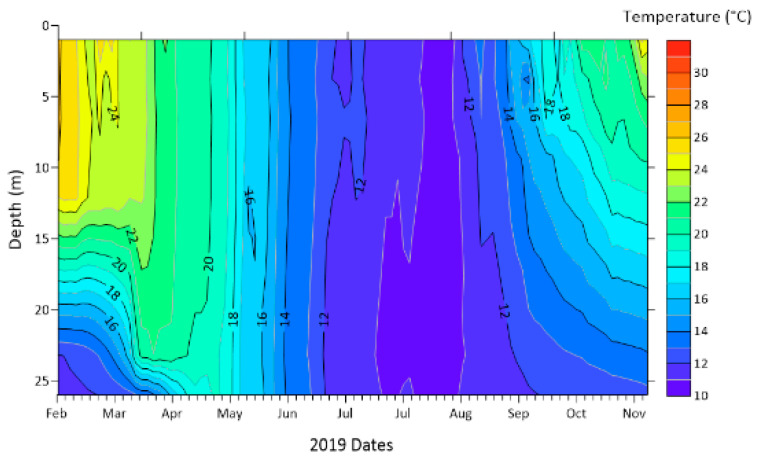
Temperatures in Hunter Lake 1 (February–November 2019) as measured by in situ continuous monitoring chains.

The sensor chain in HL1 was deployed at 25 m depth. HL1 was thermally stratified between February and early March 2019, mixed from April to September, and re-stratified in early October/November 2019 (Figure 3). During stratification, the epilimnion extended to 12 m, however the ‘hypolimnion’ was indistinct in having a pronounced temperature gradient rather than a consistent temperature after approximately 20 m. Temperatures during both stratification events ranged from a high of 27 °C at the surface to a low of 12 °C at the bottom, with bottom waters increasing briefly to a high of 18 °C on mixing.

**Figure 4:**
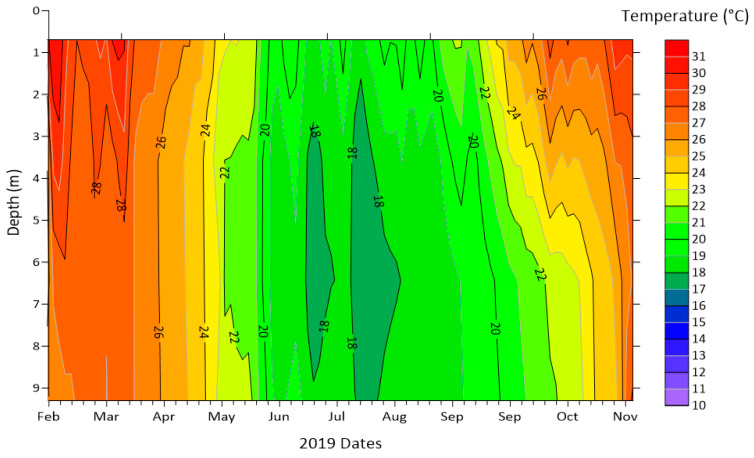
Temperatures in Bowen Lake 2 (February–November 2019) as measured by in situ continuous monitoring chains.

BL2 (maximum 9 m depth) was thermally stratified for most of 2019 with the exceptions of late April and late May when the lake appeared to mix (Figure 4). The hypolimnion formed at about 4 m and stratification was weak but consistent with a 1–2 °C difference between top and bottom. The mixing event coincided with a heavy rainfall event (March 16, 2019; 100 mm rain in 24 h) resulting in rapid cooling of surface waters and surface water runoff from highwalls.

**Figure 5:**
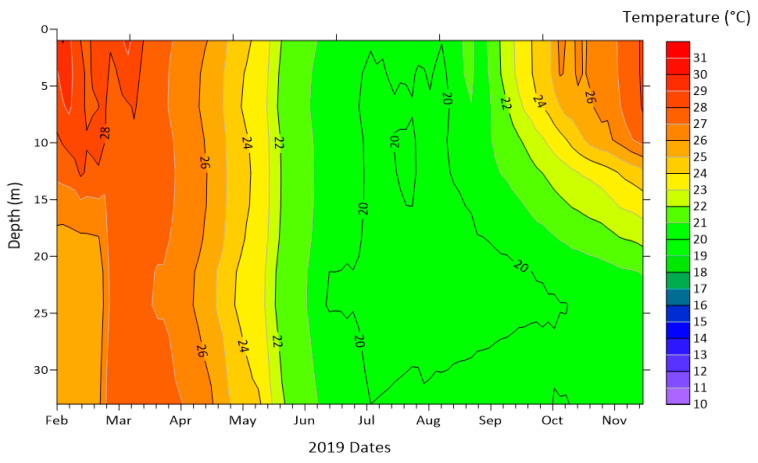
Temperatures in Bowen Lake 1 (February–November 2019) as measured by in situ continuous monitoring chains.

BL1 (maximum 33 m depth) was thermally stratified from late September to March, completely mixed from late March to June, and then weakly stratified (1–2 °C difference top to bottom) in July and August (Figure 5). The hypolimnion started at 17 m. The 100 mm rainfall event on 16 March 2019 also affected BL1 in a similar manner to BL2.

### 3.2. Water Quality

Water chemistry data (Supplementary Tables S1 and S2) were significantly different across lakes (PERMANOVA; F_(3, 23)_ = 13.3, *p* < 0.001), but not times (F_(2, 23)_ = 2.8, *p* = 0.051) or depths (F_(1, 23)_ = 0.96, *p* = 0.40). A Tukey’s post-hoc pairwise analysis indicated that water chemistry data were significantly different between most lakes with the exception of BL1 × HL1 (t = 3.25, *p* = 0.0495) and BL2 × HL1 (t = 2.89, *p* = 0.05).

**Figure 6:**
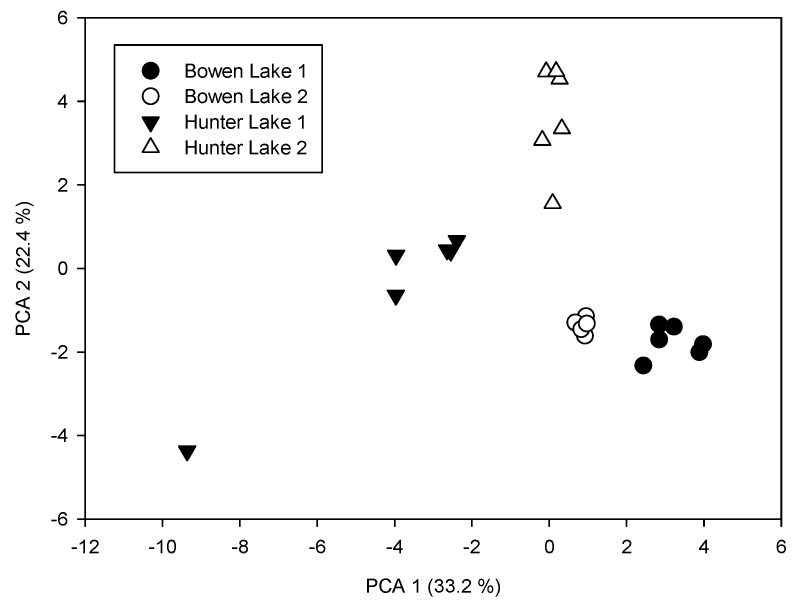
Principal component analysis (PCA) ordination of water chemistry variables from pit lakes in the Bowen Basin, Queensland and the Hunter Basin, New South Wales, Australia. Data shows an overlay of “lake” as indicated in figure legend.

A PCA plot (Axis 1; 33.2% of the variance, Axis 2; 22.4%, Axis 3; 17.1%) of water chemistry separated HL2 from the other three lakes along Axis 2 (Figure 6). Pearson–Kendall correlation analysis (|*r*|_=0.01, 22_ ≥ 0.515) indicated that PCA Axis 1 was most negatively correlated with metals (Co, Mn, K, Ni, Fe; *r* < −0.76) and most positively correlated with Cl^−^ and Na^+^ ions (r > 0.71). Axis 2 was most negatively correlated with Ba and Se (*r* < −0.69) and positively correlated with ammonium, Mg and sulfate (*r* > 0.81). In summary, water chemistry was different across lakes, but not over time or according to habitat; HL1 generally had the highest metal concentrations, BL1 had the highest concentrations of common anions, and HL2 had the highest concentrations of ammonium and sulfate (Supplementary Tables S1 and S2). Notable were the high concentrations of salinity-associated elements in lakes, particularly Na^+^ (1.07–1.76 g L^−1^) and Cl^−^ (1.58–2.71 g L^−1^) in the surface waters of BL1 (Supplementary Table S1).

**Figure 7:**
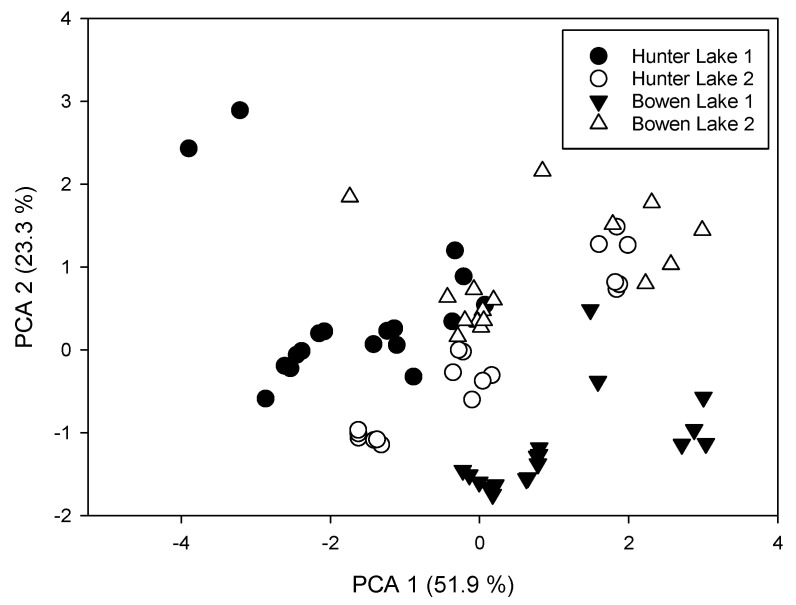
Principal components analysis (PCA) ordination of in situ water quality measurements from pit lakes in the Bowen Basin, Queensland and the Hunter Basin, New South Wales, Australia. Data shows an overlay of “lake” as indicated in figure legend.

In situ water quality measurements (Table 1) were significantly different across lakes (F_(3, 71)_ = 53.7, *p* < 0.001), times (F_(2, 71)_ = 51.0, *p* < 0.001) and depths (F_(1, 71)_ = 14.2, *p* < 0.001). Pairwise analysis indicated that in situ water quality measurements were also significantly different between all lakes and times (*p* < 0.001). A PCA (Axis 1; 51.9% of the variance, Axis 2; 23.3%, Axis 3; 14.6%) of in situ water quality measurements separated HL1 and BL1 along Axis 1, and BL1 from the other three lakes along Axis 2 (Figure 7). Pearson–Kendall correlation analysis (|*r*| = _0.01, 70_ ≥ 0.303) indicated that PCA Axis 1 was most strongly negatively associated with ORP (*r* < −0.73) and positively with pH (*r* > 0.93). PCA Axis 2 was negatively associated with conductivity (*r* < −0.71) and there were no significant positive-associated variables. In sum, in situ water quality was different across all lakes and months and between the top and bottom of the lakes. All lakes predictably warmed with warmer weather (Table 1). Relative to other lakes, BL1 tended to have higher oxygen and conductivity levels, HL1 had higher levels of ORP, and BL2 had higher temperatures and pH levels. Notable was the presence of hypoxia at the bottom of HL1 during February (1.2%, 2.5% and 80.6%) when the lake was stratified.

### 3.3. Microbial Assemblages

Pelagic microbial assemblages were significantly different across lakes (F_(3, 68)_ = 19.2, *p* < 0.001) and times (F_(2, 68)_ = 14.6, *p* < 0.001) but not between the top and bottom waters of lakes (F_(1, 68)_ = 1.13, *p* = 0.308). Pairwise analyses indicated that microbial assemblages were significantly different between all lakes (*p* < 0.001) and months (*p* < 0.01).

**Figure 8:**
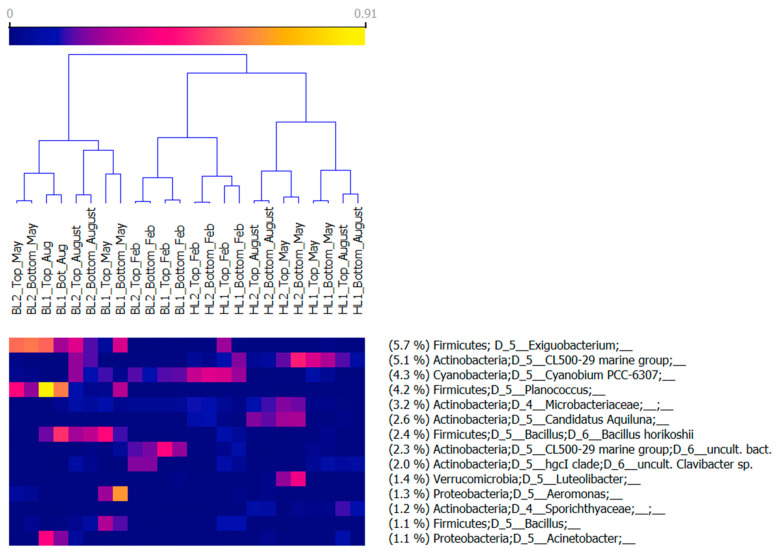
Heat map of overall most relatively abundant microbes (>1%) from pit lakes in the Bowen Basin, Queensland and the Hunter Basin, New South Wales, Australia. Heat map reflects data from most abundant replicate (*n* = 3 per lake, time and depth unless otherwise indicated in methods). Range of relative abundances per a priori group can be found in Supplementary Table S3, and the heat map split by catchment can be found in Supplementary Figure S2.

Across all lakes and times, there were 1856 unique ASV categories from 708 families of microbes, with only 13 families from domain Archaea. Most ASV were rare; 1610 ASV had a relative abundance (RA) of less than 0.01, 232 had RAs between 0.01 and 1.0, and 14 ASV had an RA of greater than 1 (Figure 8). Not only were Archaea uncommon, but they were relatively low in RA, with the most abundant Archaean (*Candidatus nitrosopumilus* sp. (Thaumarchaeota)) occupying 71st position among all lakes and times. Of the 14 taxa with RA > 1 (hereafter called ‘abundant’ taxa), SILVA resolved three of the abundant ASVs to family, 10 to genus, and one to ASV ‘species’ taxonomic level (Figure 8). The most abundant taxon was an *Exiguobacterium* (Firmicutes; 5.7% RA), followed by CL500-29 marine group (Actinobateria; 5.1% RA), *Cyanobium* PCC-6307 (Cyanobacteria; 4.3% RA), Planococcus (Firmicutes; 4.2%), Microbacteriaceae (Actinobacteria; 3.2%), Candidatus Aquiluna (Actinobacteria; 2.6%) and *Bacillus horikoshii* (Firmicutes; 2.4%), (for complete list see Figure 8). Even for the most abundant organism (*Exiguobacterium*) taxonomic representation was spatially and temporally patchy across replicates (Supplementary Table S3), times, lakes and depths. In both catchments, a ‘core’ group of relatively abundant taxa appeared to cycle over time, alternating between assemblages dominated by *Exiguobacterium* (Firmictutes) and co-domination of a Cyanobacter (*Cyanobium* PCC-6307) and Actinobacteria (Figure 8, Supplementary Table S3 and Figure S2).

Taxonomic (ASV per sample) richness (*S*) was significantly different among lakes (H_3_ = 15.1, *p* < 0.01; median rank *S* in HL1; 195, HL2; 191, BL1; 135, BL2; 134). Except for HL2 and BL1 (*p* = 0.05), Dunn’s pairwise indicated that S was significantly different (*p* < 0.05) between lakes from different catchments, where lakes from the Bowen Basin were relatively taxonomically depauperate.

Results of a RELATE procedure for microbe assemblages (weighted UniFrac distance) and in situ water quality indicated that the relationship was significant (r_s_ = 0.11, *p* < 0.01), as was that of microbe assemblages and water chemistry (r_s_ = 0.18, *p* = 0.001). Pearson–Kendall correlation analysis (|*r*| = _0.01, 60_ ≥ 0.330) indicated that on Axis 1 of an NMDS for weighted UniFrac distance (Supplementary Figure S1, 2D stress = 0.11), in situ ORP was significant (|r| = 0.355), and on NMDS Axis 2, in situ percent oxygen was significant (|r| = 0.374). Ammonium, U, sulfate, Mg and Ba were significantly co-correlated with both NMDS Axes 1 and 2 (all |r| > 0.340). DOC (|r| = 0.332) and FRP (|r| = 0.436) were significantly correlated with UniFrac NMDS Axis 1 and Al, Sr and B (all |r| ≥ 0.350) were significantly correlated with UniFrac NMDS Axis 2. 

The aforementioned NMDS for weighted UniFrac distance was not generally useful for data interpretation (Supplementary Figure S1). However, HL2 did cluster sufficiently in 2-dimensional space to indicate that, relative to other lakes, microbe distance data were correlated with higher concentrations of sulfate, Mg, ammonium and B, which appeared to drive the pattern described above.

## 4. Discussion

Pit lakes are new aquatic ecosystems of the Anthropocene [5,9,10] and occur on every inhabited continent [1]. The overall goal of this research was to determine the environmental drivers of pelagic microbe assemblages in Australian coal mine pit lakes. With the exception of Hunter Lake 2, the lakes sampled in the study were morphologically similar with nearly vertical rock walls and little to no riparian vegetation. Lakes were either neutral or slightly alkaline and could be considered mildly to moderately saline inland waters. BL1 was the most saline waterbody with an EC over 10 mS cm^−1^, followed by HL2 at 6.4 mS cm^−1^ with both HL1 and BL2 similar at 3.2–3.5 mS cm^−1^. Two of the lakes (BL1 and BL2) were located only 1 km apart. Nevertheless, all lakes had significantly different water chemistries and measurements of in situ water quality. In situ measurements were also different over time and at the surfaces and bottoms of lakes. Lakes also varied in terms of thermal mixing regime and presumed responses to precipitation. It is therefore unsurprising that this deceptively complex aquatic ecosystem could host equally complex pelagic microbial assemblages.

The use of thermistor chains to collect data on lake stratification and mixing is an essential tool in limnology, particularly for understanding the effects of climate change [39]. Few studies on pit lakes have used thermistor chains long-term. These chains collect data on a fine temporal scale and capture mixing events as they occur. Pit lakes are generally not as subject to wind mixing as natural lakes of this size and shape due to the physical protection afforded by their high rock walls [40]. However, reduced wind mixing might have accounted for the slight temperature gradients often seen in the epilimnions of deep-water lakes HL1, BL1 and BL2 that persisted for months after the establishment of stratification.

In the southern hemisphere at approximately 450 m ASL, HL1 could be considered a ‘cold’ monomictic lake, with observable summer stratification and winter mixing. BL1 and BL2 were warm monomictic lakes, where very warm waters stratified with only slight differences in temperature. The rock walls and vehicle access ramps acted as funnels to channel cool surface waters from rainfall at high-speed, forcing rapid thermal mixing in BL1 and BL2. Only one major rainfall event (100 mm) was recorded while the instrument chains were in use, yet in both BL1 and BL2 the event appeared to have resulted in lake mixing. Smaller rainfall events (< 50 mm) did not have any noticeable impact on stratification. Lake morphology and intense precipitation events likely influenced the timing of thermal mixing in pit lakes [41]. The duration and timing of thermal stratification and mixing is a known influence on lacustrine ecology [42,43], although our methods did not specifically target stochastic events.

Water chemistry was significantly different across lakes, but not times or at the top or bottom of lakes. This is in stark contrast to (for example) the Iberian Pyrite Belt acidic pit lakes of Spain [44]. The chemistry of pit lakes strongly reflects the surrounding geology [45], which would not have been expected to change substantially over the course of one year in the study lakes. HL1 tended to have high metal concentrations, BL1 had the highest concentrations of common anions, and HL2 had the highest concentrations of ammonium and sulfate. The active use of HL1 for water management at the site resulted in regular inflows and outflows, and the metals recorded in high concentrations were typical of the area and were probably introduced through pumping. High ammonium concentrations are not uncommon in pit lakes, as it is a residual from blasting with ammonium nitrate fuel oil ‘ANFO’ [46].

In contrast to water chemistry, in situ water quality was significantly different across lakes and times and at the top and bottom of lakes, and all lakes and months were significantly different from each other. BL1 had higher oxygen and conductivity levels, HL1 had higher levels of ORP, and BL2 had higher temperatures and pH levels. Additionally, the bottom layer of HL1 was hypoxic during February when water temperatures were at their warmest. Hypoxia in the hypolimnion of HL1 did not result in solubilisation of potentially harmful metals or release of nutrients as noted for acidic pit lakes [44].

Microbe assemblages were not significantly different between the surfaces and bottoms of lakes, although they were different across lakes and times, and correlated significantly with physico-chemical variables. We can consider this a ‘true’ result, rather than one due to handling error given that in the same lake, surface samples were collected by hand and bottom samples were collected using a pump. Essentially, in-lake water chemistry and water quality variability were not important enough to overcome vertical migration or drift of pelagic taxa, particularly during mixing events. As the lakes age and organic matter accumulates it is expected that like HL1, the hypolimnion will become routinely anoxic; in BL1 where the lake is stratified for the majority of the year, microbial communities could diverge between top and bottom waters and become more diverse.

As discussed above, most ASV were rare, even in the case of the most abundant organism in the study (*Exiguobacterium*) which was spatially and temporally patchy, not only among lakes, but among replicates. Species richness (or ASV richness) aligned according to catchment, where microbial assemblages in the Bowen Basin were more depauperate than those in the Hunter Basin. Geography appeared also to affect ASV diversity, with assemblages first clustering by basin, then lake, then time. While determination of biogeography was not one of the aims of this study per se, we found that microorganisms did sort geographically although more targeted research is needed [47,48,49].

Similar to other freshwater ecosystems [50,51,52], study lakes were dominated by a ‘core’ group of relatively abundant taxa that exhibited temporal turnover. *Exiguobacterium* (Firmicutes) was the most overall relatively abundant taxon in the study (5.7%) and was collected from a majority of lakes and times with the exception of February samples from BL1 bottom and BL2 top and bottom. In the Bowen study lakes, where *Exiguobacterium* was absent in February, the community was dominated by a Cyanobacter (*Cyanobium* PCC-6307) and Actinobacteria (CL500-29 marine group_uncultured bacterium, hgcI clade_uncultured *Clavibacter* sp.). In the Hunter study lakes, *Exiguobacterium* was sampled at relatively high abundances in only one sample (HL1; top, February) and unlike the Bowen Basin did not dominate microbial communities. However, the same pattern in both catchments was observed; where *Exiguobacterium* was absent in the Hunter lakes, the Actinobacters and a Cyanobacterium co-dominated the community.

The most cosmopolitan and abundant taxon (*Exiguobacterium*) is hardy and capable of primary microbial colonisation by outcompeting co-occurring taxa upon re-wetting of river sediments [53], which may be a useful trait in the ‘new ecosystem’ of a pit lake. As described above, when *Exiguobacterium* was absent or in lower abundances, *Cyanobium* PCC-6307 (Cyanobacteria) tended to dominate. *Cyanobium* PCC-6307 is a freshwater picocyanobacterium [54] that produces compounds inhibiting the growth of co-occurring bacteria, which would likely be a competitive advantage [55]. Further, neutral water quality conditions in study lakes were generally favourable to cyanobacterial growth. In extremely acidic (pH 2–3) German mine lakes, cyanobacteria were absent, and as pH increased (3–4, then 6–7), cyanobacteria became more abundant [56]. Cyanobacteria were the most abundant and diverse taxonomic group in a set of neutral mine pit lakes in Poland (pH 8) [57]. Actinobacteria (CL500-29 marine group_uncultured bacterium and hgcI clade) co-cycled in microbial planktonic communities in our study lakes; a pattern that has been previously documented in Polish lakes artificially heated with power plant discharge [58], in mesocosms [59], in the Baltic Sea [60], in an Indian brackish lagoon [61], a sewage-affected river in China [62] and a variety of other aquatic habitats both natural and artificial [63,64,65]. It is unclear why these two particular taxa co-dominate assemblages and cycled together over time and did so under such a wide variety of conditions in many different aquatic habitats. Future research in controlled laboratory experiments may assist with elucidating community assembly rules particularly regarding turnover in response to water quality and interspecific competition [66,67].

We determined the environmental drivers of pelagic microbe assemblages by first testing if ‘environment’ was significant, and which variables were most highly correlated with microbial assemblages. Both in situ water quality measurements (ORP and oxygen) and water chemistry (ammonium, U, sulfate, Mg, Ba, Al, Sr, B, DOC and FRP) were significantly correlated to microbial assemblages. Relative to other study lakes, microbe distance data in HL2 drove the overall pattern and was correlated with higher concentrations of sulfate, Mg, ammonium and B. HL2 was connected to an adjacent artificial aquifer created by backfill which likely accounted for the high levels of key water quality variables. HL2 was also characterised by a profuse growth of aquatic macrophytes and fringing rushes, although we did not target plant biofilms [68] and it is therefore unclear what role the presence of aquatic plants played in pelagic assemblages.

Archea were identified in lakes, although the most abundant Archean (*Candidatus* Nitrosoarchaeum (Thaumarchaeota)) only occupied 71st position in terms of overall relative abundance. Previously, we characterised the benthic microbe assemblages in Australian acidic and metalliferous pit lakes (pH 2.7–4.0 [23]) where an Archean (Thermoprotei (Crenarchaeota)) was the seventh-most abundant taxon in the study. Unsurprisingly, in pit lakes, taxa from domain Archaea tend to be present in higher abundances in the ‘extreme’ lakes than the more neutral lakes of the present study [47].

Dissolved organic carbon in aquatic environments supports microbial abundance and diversity [69], however BL1 had the highest DOC concentrations, followed by HL2, HL1 and BL2. In marine systems the lability of DOC might be more important for microbial diversity than the concentration [70], suggesting low-labile DOC is present in BL1. In HL2, microbe distance data were correlated with higher concentrations of sulfate, Mg, ammonium and B, which appeared to drive the pattern in microbial assemblages. HL2 had a rehabilitated catchment, fringing vegetation and abundant submerged macrophytes, with occasionally moderately high concentrations of phosphorus. Other aquatic taxa in pit lakes such as macroinvertebrates have diversities below that which would be expected given the water quality, which is likely due to a lack of suitable habitat, riparian vegetation and sufficiently available DOC [23]. As microbial communities in HL2 appeared to be correlated with chemical parameters and habitat complexity (or ‘habitat templet;’ [71,72]) was not quantified, the impact of rehabilitation on aquatic microbial communities needs testing.

## 5. Conclusions

Mine pit lakes, while appearing simple, are deceptively complex, and not all pit lakes are ‘extreme’ environments. In this study, lakes were monomictic and the timing of mixing was influenced by high rainfall events. Microbial assemblages varied across space and time, and most taxa were rare and patchy; Archeans were not prevalent. Study lakes were dominated by a ‘core’ group of relatively abundant taxa that exhibited temporal turnover, and two taxa (CL500-29 marine group and hgcI clade) co-dominated. Lakes were circumneutral and moderately saline, which was a contrast to the highly acidic and metalliferous lakes elsewhere in the world [20,23]. This lends credence to the idea of a ‘sliding scale’ of pit lake rehabilitation, whereby the combination of factors such as water quality and lake morphology determine the ease of pit lake rehabilitation [11]. However, the neutral water qualities of the pit lakes under study also facilitated the growth of cyanobacteria, a potential concern for future rehabilitation depending on lake end use [73]. We also found that in the ‘most rehabilitated’ lake (HL2), the physical connection of the lake to an artificial aquifer created through backfilling was likely responsible for high levels of sulfate, Mg, ammonium and B. This relationship drove the overall pattern of microbe distance data in the study, reinforcing the sensitivity of microbial assemblages to environmental impact, although further studies are needed at HL2.

The use of ‘traditional’ limnological techniques combined with 16S sequencing allowed us to further understand pit lakes as ecosystems, but created avenues for future research in areas such as the effects of stochastic events, benthic hypoxia, lability of organic matter, geography, and level of rehabilitation on microbe assemblages and function. In this research we determined links between the environment and microbial assemblages which is a key first step in implementing microbes as ecological indicators in pit lakes (sensu) [24].

## Figures and Tables

**Table 1:** In situ measurements collected from Australian mine pit lakes in the Bowen Basin, QLD (Bowen Lake 1; BL1, Bowen Lake 2; BL2) and the Hunter Valley NSW (Hunter Lake 1; HL1, Hunter Lake 2; HL2) in 2019. ‘Habitat’ was bottom (bot) or top of lake waters. *n* = 3 per measurement.

			Temp. (°C)	±sd	Cond. (µS cm^−1^)	±sd	pH	±sd	ORP (mV)	±sd	Oxygen (%)	±sd
HL1	Feb	Top	25.8	0.9	2913.3	46.9	8.0	0.2	174.3	35.3	97.0	9.9
		Bot	16.8	7.9	3099.7	434.8	7.6	0.3	164.0	30.4	28.1	45.5
	May	Top	17.1	0.1	3252.3	4.7	7.8	1.0	184.7	34.6	100.3	1.4
		Bot	16.4	0.9	3281.0	28.0	8.0	0.1	177.7	4.5	94.8	1.8
	Aug	Top	10.7	0.0	3251.3	7.0	7.5	0.1	185.3	19.4	93.9	0.2
		Bot	10.6	0.0	3265.0	4.0	7.6	0.1	183.7	19.2	94.3	1.0
HL2	Feb	Top	25.1	1.1	5703.3	2079.6	8.7	0.1	38.7	13.7	104.9	6.1
		Bot	25.2	0.1	6872.7	13.4	8.7	0.1	32.0	12.5	93.7	7.3
	May	Top	17.9	0.2	6877.7	2.3	8.6	0.0	151.0	13.7	101.8	0.8
		Bot	16.5	0.0	6853.0	14.2	8.6	0.0	148.3	10.2	90.3	2.0
	Aug	Top	14.9	0.4	6618.7	1.5	7.9	0.0	199.7	2.5	105.0	0.9
		Bot	13.0	0.0	6567.3	1.2	7.9	0.0	200.3	2.5	100.9	0.9
BL1	Feb	Top	30.0	0.3	10269.0	26.9	9.6	0.1	124.0	30.5	112.9	0.4
		Bot	26.6	1.0	10043.3	18.1	9.2	0.4	111.7	19.0	95.9	28.5
	May	Top	24.5	0.0	10559.0	20.9	8.5	0.0	181.3	9.1	103.0	0.3
		Bot	24.3	0.3	10534.7	6.7	8.5	0.0	163.3	3.8	100.7	0.6
	Aug	Top	21.4	0.5	10506.3	16.2	8.3	0.0	183.7	4.0	106.4	0.8
		Bot	19.7	0.2	10413.7	12.1	8.2	0.0	183.7	4.0	100.4	4.0
BL2	Feb	Top	30.0	0.2	3545.0	3.0	9.6	0.1	81.7	32.0	109.8	1.4
		Bot	26.7	0.2	3547.3	90.9	9.3	0.2	82.3	27.5	80.9	20.7
	May	Top	22.2	0.1	3227.7	4.7	8.7	0.0	169.3	5.0	99.3	0.5
		Bot	22.0	0.1	3225.7	0.6	8.7	0.0	165.0	5.6	98.6	1.0
	Aug	Top	19.8	0.4	3420.0	11.3	8.4	0.0	140.7	23.2	104.2	0.8
		Bot	18.5	0.1	3402.0	20.8	8.3	0.2	139.7	23.0	78.2	32.5

## Data Availability

The data presented in this study are available on request from the corresponding author.

## Supplementary Materials

The following are available online at https://www.mdpi.com/article/10.3390/microorganisms9061207/s1, Table S1: Results of water chemistry analyses collected from Australian mine pit lakes (1/2), Table S2: Results of water chemistry analyses collected from Australian mine pit lakes (2/2), Table S3: Heat map of overall most abundant (>1%) pelagic microbes collected from mine pit lakes in Australia in 2019, Figure S1: Nonmetric multidimensional scaling (NMDS) ordination of pelagic microbe assemblages from pit lakes in the Bowen Basin, Queensland and the Hunter Basin, New South Wales, Australia.
