# Supplementary material for: Aquatic Ecosystems of the Anthropocene: Limnology and Microbial Ecology of Mine Pit Lakes
Source: Microorganisms. 2021 Jun 3;9(6):1207. doi: 10.3390/microorganisms9061207 (PMC8228816; doi:10.3390/microorganisms9061207)
Supplement: Supplementary file 1 [file microorganisms-09-01207-s001.zip › microorganisms-1224750-supplementary.pdf]

Supplementary Table 1. Results of water chemistry analyses collected from Australian mine pit lakes (1/2) in the Bowen Basin, QLD (Bowen Lake 1; BL1, Bowen Lake 2; BL2) and Hunter Valley NSW (Hunter Lake 1; HL1, Hunter Lake 2; HL2) in 2019. 'Habitat' was bottom (bot) or top of lake waters. Data in italics were below the reportable limit but above zero and thus were assigned a number equal to half the detection limit [38].

		Limit:	<0.5	<0.5	<0.05	<0.05	<0.01	<0.02	<0.05	<0.25	<0.25	<0.01	<0.05	<0.01	<0.02	<0.02	<0.5	<0.5	<0.5	<0.5	<0.5	<0.5	<0.5
		Units:	μg L^{-1}	μg L^{-1}	μg L^{-1}	μg L^{-1}	μg L^{-1}	μg L^{-1}	μg L^{-1}	μg L^{-1}	μg L^{-1}	μg L^{-1}	μg L^{-1}	μg L^{-1}	μg L^{-1}	μg L^{-1}	μg L^{-1}	μg L^{-1}	mg L^{-1}	mg L^{-1}	mg L^{-1}	mg L^{-1}	mg L^{-1}
Lake	Habitat	Date	B	Al	Cr	Mn	Fe	Co	Ni	Cu	Zn	As	Se	Cd	Pb	U	Ba	Sr	Ca	K	Mg	Na	Cl-
BL1	Bot	Feb	346.6	41.1	*0.025*	0.29	*0.005*	*0.01*	1.75	2.52	*0.125*	0.69	10.7	*0.005*	*0.01*	2.21	61.9	4793.7	26.0	12.3	104.6	1761.3	1575.3
		May	136.4	30.8	*0.025*	0.79	3.02	*0.01*	0.90	2.45	16.3	*0.005*	5.17	*0.005*	*0.01*	0.68	44.3	3050.1	26.8	6.35	59.4	1069.9	1627.9
		Aug	209.0	43.2	*0.025*	0.96	4.20	*0.01*	1.62	2.25	*0.125*	0.03	5.11	*0.005*	*0.01*	1.34	49.6	4250.5	42.9	9.17	85.5	1353.1	2711.9
	Top	Feb	171.9	42.0	*0.025*	*0.025*	*0.005*	*0.01*	0.94	0.97	*0.125*	*0.005*	5.55	*0.005*	*0.01*	0.65	47.3	3189.5	22.7	5.46	56.8	983.3	1386.7
		May	204.1	33.3	*0.025*	2.10	1.54	*0.01*	1.10	1.05	*0.125*	0.12	5.94	*0.005*	*0.01*	1.26	49.9	3764.8	29.3	8.50	80.4	1267.2	1065.2
		Aug	257.7	35.0	*0.025*	2.15	13.9	*0.01*	1.54	2.46	*0.125*	0.22	7.08	*0.005*	*0.01*	2.40	64.6	5552.8	41.6	11.5	104.3	1629.1	1621.9
BL2	Bot	Feb	200.5	36.1	*0.025*	3.96	1.00	*0.01*	1.55	2.20	*0.125*	0.78	4.71	*0.005*	*0.01*	1.50	76.6	1067.8	20.7	6.82	49.6	620.0	495.6
		May	116.8	28.6	*0.025*	1.23	2.48	*0.01*	0.96	1.24	*0.125*	0.30	3.64	*0.005*	*0.01*	0.85	52.6	675.2	17.7	4.46	30.5	463.4	291.3
		Aug	106.7	20.2	*0.025*	1.00	13.6	*0.01*	0.44	1.18	*0.125*	*0.005*	2.39	*0.005*	*0.01*	0.73	59.9	760.6	19.9	3.56	31.2	464.9	690.4
	Top	Feb	167.4	34.5	*0.025*	0.28	4.05	*0.01*	0.70	1.60	*0.125*	0.50	3.75	*0.005*	*0.01*	0.98	69.0	890.6	17.3	4.12	35.2	500.7	301.6
		May	158.2	33.7	*0.025*	0.38	4.68	*0.01*	0.90	1.78	*0.125*	0.47	4.00	*0.005*	*0.01*	1.35	64.6	822.1	20.4	5.20	39.4	545.8	628.8
		Aug	155.9	24.7	*0.025*	0.08	1.07	*0.01*	0.74	1.86	*0.125*	0.34	3.64	*0.005*	*0.01*	1.56	67.6	948.1	22.5	5.47	43.3	620.6	359.6
HL2	Bot	Feb	519.7	74.6	*0.025*	26.3	16.5	*0.01*	1.39	2.55	*0.125*	0.27	*0.025*	*0.005*	*0.01*	5.54	14.6	1879.4	51.2	25.4	291.3	1017.8	450.1
		May	454.4	40.3	*0.025*	38.0	31.2	*0.01*	2.31	2.31	*0.125*	0.19	*0.025*	*0.005*	*0.01*	8.55	13.5	3458.2	113.9	29.6	304.5	1019.5	188.2
		Aug	227.3	17.8	*0.025*	57.2	6.47	*0.01*	1.58	0.57	*0.125*	*0.005*	*0.025*	*0.005*	*0.01*	5.36	7.32	2644.4	105.9	15.7	184.5	823.8	458.4
	Top	Feb	240.9	47.8	*0.025*	10.9	18.2	*0.01*	1.42	2.47	*0.125*	*0.005*	*0.025*	*0.005*	*0.01*	1.89	7.00	1153.9	25.2	12.3	155.5	677.9	457.5
		May	449.0	30.1	*0.025*	43.7	18.9	*0.01*	1.93	1.26	*0.125*	0.19	*0.025*	*0.005*	*0.01*	8.10	13.1	3731.4	122.5	29.2	304.5	1059.7	293.2
		Aug	356.0	23.3	*0.025*	75.4	8.43	*0.01*	2.37	1.01	*0.125*	*0.005*	*0.025*	*0.005*	*0.01*	8.62	8.21	3308.1	135.2	25.6	286.1	990.3	423.7
HL1	Bot	Feb	73.7	55.3	*0.025*	868.8	23.2	2.91	39.2	11.4	9.72	*0.005*	0.41	*0.005*	*0.01*	*0.01*	40.6	898.8	132.1	51.91	111.91	317.7	74.3
		May	42.0	40.1	*0.025*	222.0	25.3	1.88	37.6	1.62	2.17	*0.005*	*0.025*	*0.005*	*0.01*	*0.01*	30.8	832.9	151.6	44.5	96.2	266.6	171.2
		Aug	29.2	18.9	*0.025*	497.1	6.59	3.31	33.8	0.93	1.70	*0.005*	*0.025*	*0.005*	*0.01*	*0.01*	33.0	772.6	193.4	43.4	93.1	260.8	182.4
	Top	Feb	58.5	66.5	3.14	686.7	64.2	4.87	33.6	28.1	10.3	2.61	3.49	1.79	2.29	2.55	31.0	766.4	137.6	41.3	89.9	245.3	91.1
		May	45.7	30.1	*0.025*	140.0	16.8	1.60	44.9	1.53	*0.125*	*0.005*	*0.025*	*0.005*	*0.01*	*0.01*	34.6	878.7	162.1	49.8	105.3	345.2	108.6
		Aug	44.3	22.6	*0.025*	808.1	7.21	6.31	55.4	0.58	3.71	*0.005*	*0.025*	*0.005*	*0.01*	*0.01*	41.7	893.6	204.3	61.9	130.0	381.50	183.2

Supplementary Table 2. Results of water chemistry analyses collected from Australian mine pit lakes (2/2) in the Bowen Basin, QLD (Bowen Lake 1; BL1, Bowen Lake 2; BL2) and Hunter Valley NSW (Hunter Lake 1; HL1, Hunter Lake 2; HL2) in 2019. 'Habitat' was bottom (bot) or top of lake waters. Data in italics were below the reportable limit but above zero and thus were assigned a number equal to half the detection limit [38].

		Limit:	< 3	< 2	< 50	< 2	< 0.5	< 0.5
		Units	µg L^{-1}	µg L^{-1}	µg L^{-1}	µg L^{-1}	mg L^{-1}	mg L^{-1}
Lake	Habitat	Date	NH_4	NOx	TN	FRP	SO_4^{2-}	DOC
BL1	Bot	Feb	1.5	1800.0	1860.0	*1.0*	488.1	6.02
		May	16.3	1520.0	1680.0	*1.0*	454.2	55.3
		Aug	*1.5*	1590.0	1710.0	*1.0*	675.0	174.9
	Top	Feb	*1.5*	1720.0	1730.0	3.53	293.6	2.52
		May	*1.5*	726.5	1900.0	*1.0*	431.0	28.17
		Aug	*1.5*	1320.0	1390.0	*1.0*	454.1	8.234
BL2	Bot	Feb	13.1	*1.0*	148.0	*1.0*	108.9	2.67
		May	*1.5*	2.43	109.0	*1.0*	79.5	4.52
		Aug	40.2	12.9	342.0	*1.0*	140.7	41.9
	Top	Feb	*1.5*	*1.0*	109.4	4.06	70.3	2.62
		May	*1.5*	4.55	96.9	*1.0*	129.9	2.95
		Aug	*1.5*	*1.0*	199.0	*1.0*	82.1	3.17
HL2	Bot	Feb	*1.5*	3.92	249.0	6.57	1356.3	9.47
		May	216.0	4.2	323.0	2.41	2049.7	38.0
		Aug	187.0	3.74	336.0	*1.0*	1996.0	7.17
	Top	Feb	9.16	*1.0*	1010.0	5.57	1457.8	22.0
		May	202.0	3.16	400.0	2.77	1990.1	7.85
		Aug	196.0	3.0	330.0	*1.0*	2034.3	7.08
HL1	Bot	Feb	*1.5*	10.6	280.0	2.35	629.8	4.32
		May	27.5	*1.0*	182.0	*1.0*	1017.8	2.84
		Aug	23.1	29.0	107.0	*1.0*	1056.0	2.72
	Top	Feb	*1.5*	20.2	163.0	2.06	626.2	4.91
		May	*1.5*	*1.0*	133.0	3.92	922.6	2.07
		Aug	21.1	32.5	187.0	*1.0*	1030.8	2.42

Supplementary Table 3. Heat map of overall most abundant (> 1%) pelagic microbes collected from mine pit lakes in Australia in 2019. (Overall percent abundance in parentheses shown after taxa name). Taxa were numbered from first to fourteenth most relatively abundant across all lakes (BL1, BL2, HL1, HL2), habitats (Top, Bottom) and times (February, May, August). Per sample relative abundances are ranges (n = 3 unless otherwise specified in methods) and colour was consistently assigned based on sample with highest relative abundance.

Phylum; D_4__Family; D_5__Genus; D_6__Species	BL1_Top_Feb	BL1_Bottom_Feb	BL1_Top_May	BL1_Bottom_May	BL1_Top_Aug	BL1_Bot_Aug	BL2_Top_Feb	BL2_Bottom_Feb	BL2_Top_May	BL2_Bottom_May	BL2_Top_August	BL2_Bottom_August
1. Firmicutes; D_5__Exiguobacterium;__(5.7 %)	<0.01	0	<0.01-0.06	<0.01-0.32	0.04-0.56	0.04-0.24	0	0	0.37-0.57	0.31-0.59	0.03-0.33	<0.01-0.16
2. Actinobacteria;D_5__CL500-29 marine group;__ (5.1 %)	<0.01	<0.01	<0.01	<0.01	0	<0.01	<0.01	<0.01	<0.01-0.02	<0.01	0.10-0.22	0.11-0.16
3 Cyanobacteria;D_5__Cyanobium PCC-6307;__ (4.3 %)	0.01-0.16	0.01-0.17	0.01-0.15	<0.01-0.03	<0.01	<0.01	0.01-0.16	0.05-0.11	<0.01-0.03	0.02	0.10-0.22	0.10-0.12
4. Firmicutes;D_5__Planococcus;__ (4.2%)	0	0	0-0.03	0-0.27	0.03-0.91	0.04-0.60	0	0	0.16-0.41	0.08-0.22	<0.01-0.10	<0.01-0.03
5. Actinobacteria;D_4__Microbacteriaceae;__;__ (3.2 %)	0.03-0.04	0.04-0.05	0.02-0.10	<0.01-0.03	<0.01	<0.01-0.03	0.03-0.04	0.04	<0.01	<0.01	0.06-0.08	0.04-0.06
6. Actinobacteria;D_5__Candidatus Aquiluna;__ (2.6 %)	<0.01	<0.01	<0.01	<0.01	0	<0.01	<0.01	<0.01	<0.01	<0.01	<0.01	<0.01
7. Firmicutes;D_5__Bacillus;D_6__Bacillus horikoshii (2.4 %)	0	0	0-0.42	0-0.15	<0.01-0.18	0.03-0.48	0	0	0	<0.01	<0.01-0.24	0.15-0.28
8. Actinobacteria;D_5__CL500-29 marine group;D_6__uncult. bact. (2.3 %)	0-0.41	0.02-0.22	0.05-0.09	0.02-0.05	<0.01	<0.01	0.14-0.16	0.11-0.19	<0.01	<0.01	<0.01	<0.01-0.02
9. Actinobacteria;D_5__hgcI clade;D_6__uncult. Clavibacter sp. (2.0 %)	0	0	0	0	0	0	0.17-0.20	0.17-0.20	<0.01	<0.01	0.03-0.07	0.02-0.03
10.Verrucomicrobia;D_5__Luteolibacter;__ (1.4 %)	<0.01	<0.01-0.02	<0.01	<0.01	<0.01	<0.01	<0.01	0	<0.01	<0.01	<0.01	<0.01
11. Proteobacteria;D_5__Aeromonas;__ (1.3 %)	<0.01	<0.01	<0.01-0.23	0.02-0.65	0	<0.01	<0.01	<0.01	<0.01-0.06	<0.01-0.04	<0.01	<0.01
12. Actinobacteria;D_4__Sporichthyaceae;__;__ (1.2 %)	0	0	0	0	0	0	<0.01	<0.01	<0.01	0	<0.01	<0.01
13. Firmicutes;D_5__Bacillus;__ (1.1 %)	<0.01	<0.01	<0.01-0.26	<0.01-0.16	<0.01	<0.01-0.01	<0.01	0	<0.01	0-0.03	<0.01-0.03	0.03-0.07
14. Proteobacteria;D_5__Acinetobacter;__ (1.1 %)	<0.01	0	0	<0.01	0.01-0.4	0.02-0.20	0	<0.01	<0.01	<0.01	<0.01-0.06	<0.01

HL1_Top_Feb	HL1_Bottom_Feb	HL1_Top_May	HL1_Bottom_May	HL1_Top_August	HL1_Bottom_August	HL2_Top_Feb	HL2_Bottom_Feb	HL2_Top_May	HL2_Bottom_May	HL2_Top_August	HL2_Bottom_August	Legend
<0.01-0.23	<0.01-0.01	<0.01	0-0.02	<0.01	<0.01	<0.01	<0.01	<0.01	<0.01	<0.01	<0.01	0
0.04-0.11	0.09-0.20	0.24-0.32	0.12-0.26	0.10-0.16	0.05-0.07	0.05	0.03	0.12-0.17	0.15-0.45	0.03-0.04	0.05-0.06	<0.01
0.08-0.32	<0.01-0.23	0.05-0.08	0.01-0.04	0	0	0.28-0.29	0.33	<0.01	<0.01	0	0	≤0.01-0.25
<0.01	<0.01	<0.01	<0.01	<0.01	<0.01	<0.01	<0.01	0	0	<0.01	<0.01	0.26-0.50
<0.01-0.04	0-0.02	0.02	0-0.02	<0.01-0.01	<0.01	0.11-0.13	0.12	0.19-0.20	0-0.18	0.09-0.11	0.07-0.15	0.6-0.75
<0.01-0.02	0-0.01	<0.01	<0.01	<0.01-0.02	<0.01	0.09	0.12	0.19-0.24	0-0.25	0.12-0.20	0.14-0.17	>0.76
0-0.10	<0.01-0.05	<0.01	0-0.03	<0.01	<0.01	<0.01	0	0	<0.01	<0.01	<0.01	
<0.01-0.03	0.02-0.08	0.02-0.03	0.02	<0.01	<0.01	<0.01	<0.01	<0.01	<0.01	0	0	
<0.01-0.09	<0.01-0.04	<0.01-0.04	0.02-0.08	0.05-0.06	0.03-0.07	0	0	<0.01	<0.01	<0.01	<0.01	
<0.01	0	0	0	<0.01	<0.01	<0.01	<0.01	0.19-0.22	0.08-0.36	<0.01	0.01-0.02	
<0.01	<0.01-0.02	<0.01	<0.01	<0.01	<0.01	<0.01	<0.01	<0.01	<0.01	<0.01	<0.01-0.02	
<0.01	<0.01	<0.01-0.02	0.02	0.11-0.15	0.05-0.10	0	0	<0.01	0.01-0.03	0.06-0.08	0.05-0.07	
<0.01-0.11	<0.01-0.12	<0.01	<0.01	<0.01	<0.01	<0.01	<0.01	0	<0.01	0	0	
0	<0.01	<0.01	<0.01	<0.01-0.10	<0.01	0	0	<0.01	<0.01	<0.01	<0.01-0.03	

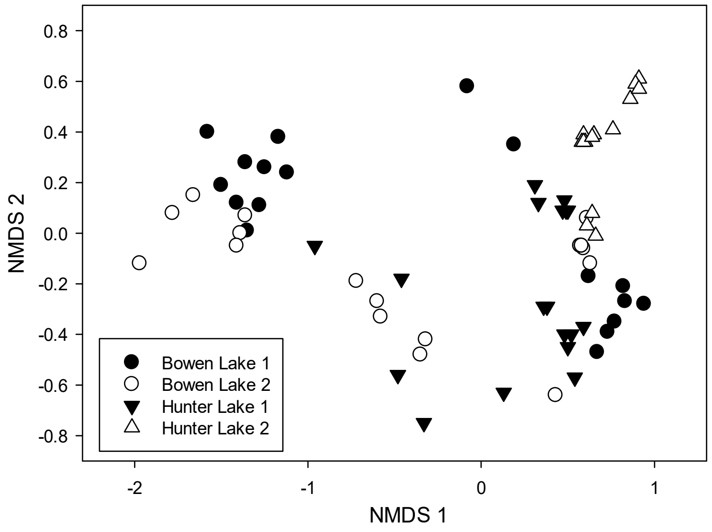

Supplementary Figure 1. Nonmetric multidimensional scaling (NMDS) ordination of pelagic microbe assemblages from pit lakes in the Bowen Basin, Queensland and Hunter Basin, New South Wales, Australia. Data shows an overlay of "lake" as indicated in figure legend.

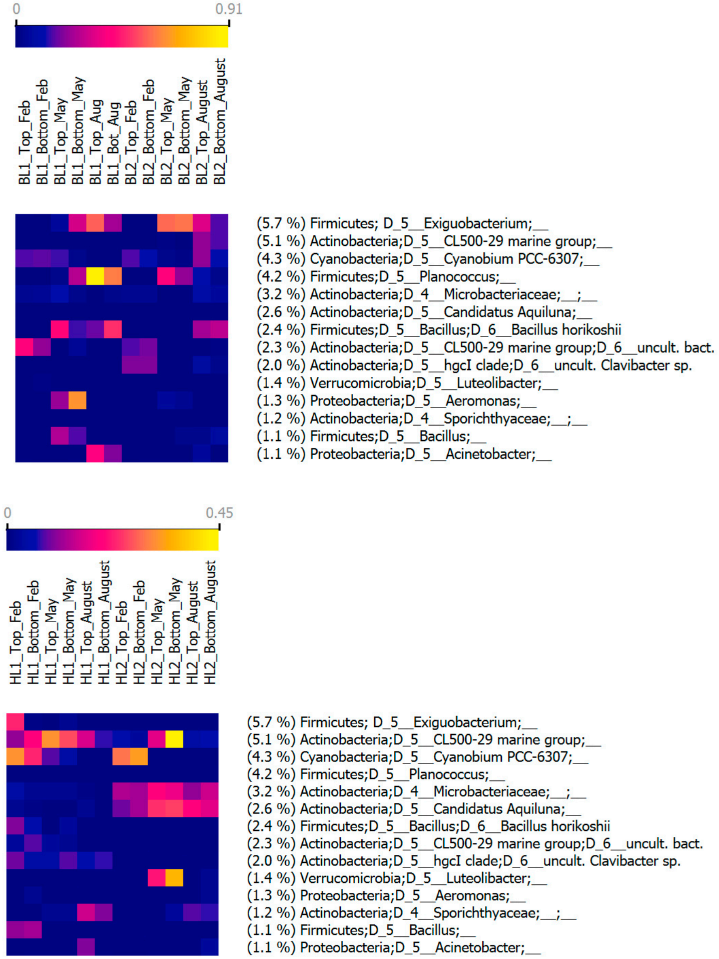

Supplementary Figure 2. Heat map of overall most relatively abundant microbes (> 1%) from pit lakes in the Bowen Basin, Queensland (top) and Hunter Basin, New South Wales (bottom), Australia. Heat map is composed of the same data as Figure 8 but divided here by catchment to facilitate understanding of temporal cycling of 'core' microbial taxa; *Exiguobacterium* (Firmicutes) vs. Actinobacteria and a Cyanobacterium.
